# Effect of Body Size on Plasma and Tissue Pharmacokinetics of Danofloxacin in Rainbow Trout (*Oncorhynchus mykiss*)

**DOI:** 10.3390/ani14223302

**Published:** 2024-11-16

**Authors:** Kamil Uney, Duygu Durna Corum, Pedro Marín, Devran Coskun, Ertugrul Terzi, Elena Badillo, Orhan Corum

**Affiliations:** 1 Department of Pharmacology and Toxicology, Faculty of Veterinary Medicine, University of Selcuk, Konya 42031, Türkiye; kuney@selcuk.edu.tr; 2 Department of Pharmacology and Toxicology, Faculty of Veterinary Medicine, University of Hatay Mustafa Kemal, Hatay 31060, Türkiye; ddurnacorum@gmail.com (D.D.C.); orhancorum46@hotmail.com (O.C.); 3 Department of Pharmacology, Faculty of Veterinary Medicine, University of Murcia, 30100 Murcia, Spain; ebp2@um.es; 4 Department of Pharmacology and Toxicology, Faculty of Veterinary Medicine, University of Siirt, Siirt 56100, Türkiye; devrancoskun@gmail.com; 5 Department of Veterinary Medicine, Devrekani TOBB Vocational School, University of Kastamonu, Kastamonu 37200, Türkiye; ertugrulterzi@gmail.com

**Keywords:** antibiotics, aquaculture, body size related pharmacokinetics, HPLC, fluoroquinolones

## Abstract

Danofloxacin is a fluoroquinolone antibiotic approved for use in fish. It can be used for bacterial infections in fish of all body sizes. However, physiological differences in fish depending on size may change the pharmacokinetics of danofloxacin and therefore its therapeutic efficacy. In this study, the change in the pharmacokinetics of danofloxacin in rainbow trout of various body sizes was revealed for the first time. The objective of this investigation was to compare the plasma and tissue pharmacokinetics of danofloxacin in rainbow trout of different body sizes. The study was conducted at 14 ± 0.5 °C in fish of small, medium, and large body size and danofloxacin was administered orally at a dose of 10 mg/kg. Concentrations of this antimicrobial in tissues and plasma were quantified by high performance liquid chromatography with ultraviolet detector. The plasma elimination half-life (t_1/2ʎz_), volume of distribution (V_darea_/F), total clearance (CL/F), peak concentration (C_max_), and area under the plasma concentration–time curve (AUC_0–last_) were 27.42 h, 4.65 L/kg, 0.12 L/h/kg, 2.53 µg/mL, and 82.46 h·µg/mL, respectively. Plasma t_1/2ʎz_, AUC_0–last_ and C_max_ increased concomitantly with trout growth, whereas CL/F and V_darea_/F decreased. Concentrations in liver, kidney, and muscle tissues were higher than in plasma. C_max_ and AUC_0–last_ were significantly higher in large sizes compared to small and medium sizes in all tissues. The scaling factor in small, medium, and large fish was 1.0 for bacteria with MIC thresholds of 0.57, 0.79, and 1.01 µg/mL, respectively. These results show that therapeutic efficacy increases with body size. However, since increases in danofloxacin concentration in tissues of large fish may affect withdrawal time, attention should be paid to the risk of tissue residue.

## Simple Summary

Changes in body size in fish can alter the pharmacokinetics of drugs, thereby affecting their therapeutic efficacy. Fish are among the animals that exhibit the greatest variability in body size during their life cycle. Despite being exposed to bacterial infections at all stages of life, the dosage regimens of antibacterial drugs have not been adjusted for age/body mass changes. The plasma and tissue pharmacokinetics of danofloxacin differed according to rainbow trout size. Pharmacokinetic/pharmacodynamic findings suggest that treatment efficacy is positively correlated with body size. However, in natural infections caused by susceptible bacteria, pharmacokinetic/pharmacodynamic studies in different fish sizes are needed to define optimal dosing regimens.

## 1. Introduction

Türkiye is one of the most important trout farming countries in the world. Trout account for 37.2% of the country’s fish production and 23% of its aquaculture income. Rainbow trout (*Oncorhynchus mykiss*) is economically valuable because of its rapid growth, tolerance to high temperatures, and suitability for hatchery culture [1]. Bacterial infections are common in fish, including trout, and cause significant economic losses and mortality [2]. Fish health is directly affected by physicochemical changes in the aquatic environment, such as temperature and salinity, which facilitate the emergence and spread of disease [2]. Therefore, antibiotics are widely used to prevent or treat bacterial infections in fish [3]. The increased use of antibiotics and lack of attention to the appropriate dosage regimen is leading to an increase in bacterial resistance, and to prevent this, it is important to use drugs in appropriate dosage regimens [3,4].

Fluoroquinolones are widely used to treat bacterial infections in fish due to their broad spectrum of activity, efficacy against most fish pathogens at low concentrations, and post-antibiotic effects [5]. Danofloxacin is a type of fluoroquinolone antibiotic that is an inhibitor of the bacterial enzymes DNA-gyrase and topoisomerase IV. It is active against both Gram-negative and Gram-positive bacteria [6]. Danofloxacin has superior pharmacokinetic properties, including a long elimination half-life, high bioavailability, and a high volume of distribution, compared with enrofloxacin, another well-known member of the fluoroquinolone group [7,8,9]. Danofloxacin is approved in the European Union for the treatment of ulcers, septicemia, and skin infections in fish species [10] and is generally recommended at a dose of 10 mg/kg [11].

Age-related physiological changes in plasma protein concentration, enzyme capacity, body water/fat ratio, and organ maturation may affect the absorption, distribution, metabolism, and excretion of drugs [12,13,14]. Fish are among the vertebrates with the most variable body mass ratios throughout their life cycle and, being heterotherms, their growth and development are influenced by environmental factors [15]. Therefore, it is more appropriate to assess life cycles based on body size rather than age [16]. Body composition (water, fat, and protein), organ weights, and metabolism of fish vary with body size [17,18,19]. These physiological differences may affect drug pharmacokinetics, therapeutic efficacy, and treatment success. Fluoroquinolones are critical antibiotics in human medicine and the success of danofloxacin treatment will also affect the development of bacterial resistance [20]. Therefore, it is not appropriate to use the same dosing regimen for bacterial diseases in fish of different sizes and studies should be conducted on the target fish body size.

Although the body distribution of some toxic substances in fish has been shown to vary with body size [19,21,22], studies on antibiotics are limited. A recent study showed that the pharmacokinetic characteristics of oxytetracycline, including peak concentration, area under the plasma concentration versus time curve, volume of distribution, and clearance, varied with rainbow trout size [16]. Danofloxacin pharmacokinetics and clearance studies have been demonstrated in various fish species [5,11,20,23,24,25,26]. The pharmacokinetics of danofloxacin showed significant differences in these studies and it has been suggested that these differences may be due to species, water temperature and size differences [5]. However, to the best of our knowledge, there are no studies demonstrating the pharmacokinetic changes of danofloxacin as a function of fish body size. The aim of this study was to determine the changes in plasma and tissue (muscle, liver, and kidney) pharmacokinetics following oral administration of danofloxacin at a dose of 10 mg/kg in small, medium, and large rainbow trout.

## 2. Materials and Methods

### 2.1. Fish

The study was conducted at a local fish farm using a total of 324 healthy rainbow trout of small (*n* = 108, 25.47 ± 2.58 g), medium (*n* = 108, 106.19 ± 3.81 g), and large (*n* = 108, 227.30 ± 6.34 g) body size (mean ± SD). Fish that had not been medicated in the one month prior to the study and showed no signs of disease or injury were included in the study. The fish were kept in concrete ponds with a constant flow of spring water at a temperature of 14 ± 0.5 °C and a pH of 8.0 ± 0.2 under natural daylight conditions. Three ponds of 36 fish each were used for each size group. To facilitate their adaptation to the environment, the fish were transferred to the ponds two weeks before the start of the study. The fish were fed pelleted diets at a daily rate of 2% of body weight but were fasted for 12 h before and after the administration of the drugs to reduce the influence of the dietary content on the absorption of danofloxacin. The study received approval (2020/21) by the Animal Experiments Local Ethics Committee of Kastamonu University (Kastamonu, Turkey).

### 2.2. Experimental Design

To reduce traumatic injury and stress in trout, drug administration and sampling (plasma and tissues) were performed under tricaine methanesulfonate anaesthesia (MS-222) using six different fish at each sampling time. For drug administration to the fish, the commercial preparation of danofloxacin (Advocin, 25 mg/mL, solution for injection, Zoetis, Istanbul, Türkiye) was diluted with injection water at a concentration of 1 mg/mL (for small body size) and 10 mg/mL (for medium and large body size). A total of 324 fish were randomly assigned to three groups based on body size: small (*n* = 108), medium (*n* = 108), and large (*n* = 108). All groups received a dose of 10 mg/kg danofloxacin orally by gavage. Blood samples (0.5–2 mL) were collected from the caudal vessel into heparinised anticoagulant tubes under anaesthesia (MS-222, 200 mg/L) at 0 (control), 0.25, 0.5, 1, 2, 4, 8, 12, 24, 48, 72, 96, 120, 144, 168, 192, 216, and 240 h. In addition, fish were euthanised with high dose anaesthesia (MS-222, 300 mg/L) at these sampling times and muscle, liver, and kidney samples were collected. Plasma was obtained by centrifugation of blood samples at 4000× *g* for 10 min and stored with tissue samples at −80 °C until analysis.

### 2.3. Danofloxacin Analysis

HPLC-UV was used to analyse danofloxacin in plasma and tissue samples according to previously published procedures [19,27]. Briefly, tissues were homogenised using a homogeniser at 10,000 rpm for 30 s. A total of 100 mg of tissue and 100 μL of plasma were transferred to 2 mL microcentrifugation vials and 200 μL of acetonitrile was added. This mixture was vortexed for 30 s and then centrifuged at 10,000× *g* for 10 min. Then, 100 µL of the supernatant was transferred to new tubes and 100 µL of water was added and vortexed for 5 s. The mixture was transferred to autosampler vials and 20 µL was injected into the HPLC system. The HPLC system consists of a pump, column oven, degasser, autosampler, and UV-VIS detector. Separation was performed on an InertSustain C18 analytical column (4.6 × 250 mm; 5 μm) maintained at 40 °C. The UV detection wavelength was set at 280 nm. The mobile phase consisted of 18% acetonitrile and 82% solution (0.4% triethylamine + 0.4% orthophosphoric acid) using the isocratic method at a flow rate of 1 mL/min.

A stock solution of danofloxacin (≥98%, Sigma-Aldrich, St. Louis, MI, USA) was prepared in purified water to achieve a concentration of 200 μg/mL. Stock solution was diluted to prepare working standards. The calibration standards and quality control samples were prepared by mixing blank plasma and tissue with danofloxacin working standard solutions. The calibration curve of danofloxacin for plasma and tissue was linear (R^2^ > 0.9987) between 0.04 and 40 μg/mL (g). Quality control samples of danofloxacin at concentrations of 0.1 μg/mL (g), 1 μg/mL (g) and 10 μg/mL (g) were used to assess recovery, precision, and accuracy. The recovery of danofloxacin in plasma and tissue was >93% and >89%, respectively. The lower limit of quantitation (LLOQ) for danofloxacin in rainbow trout plasma and tissue was 0.04 μg/mL (g), with a coefficient of variation of less than 20% and a bias of ±15%. The intraday and interday coefficients of variation were ≤7.15% and ≤8.43%, respectively. The intraday and interday biases were ±4.39% and ±3.03% respectively.

### 2.4. Pharmacokinetic Analysis

Plasma and tissue concentration-time data were analysed using WinNonlin 6.1. software with non-compartmental pharmacokinetic analysis. Pharmacokinetic parameters of danofloxacin were determined from mean plasma and tissue concentrations at sampling times, as different fish were used at each sampling time, as documented in previous studies [28,29]. The peak concentration (C_max_) and the time to reach C_max_ (T_max_) were directly obtained from the data. The volume of distribution (V_darea_/F), total body clearance (CL/F), terminal elimination half-life (t_1/2λz_), mean residence time (MRT), area under the plasma concentration versus time curve (AUC), and the AUC extrapolated from tlast to ∞ in % of the total AUC (AUC_extrap_%) were calculated.

### 2.5. Data Analysis

Plasma and tissue concentrations are presented as mean ± standard deviation. When different animals are used at each sampling time, it is recommended that pharmacokinetic calculations be performed on mean concentrations [28,29]. In this case, as each group is considered as a single animal, only one pharmacokinetic parameter is obtained. Pharmacokinetic parameters calculated in this way are not compared between routes of administration using the statistical test. Therefore, statistical evaluation between groups was not possible in this study.

## 3. Results

### 3.1. Plasma Pharmacokinetic Parameters

**Figure 1:**
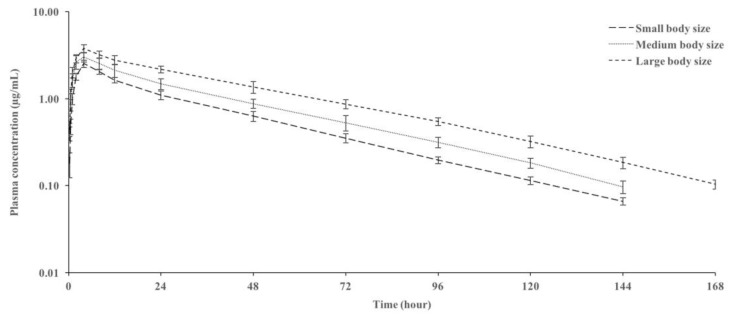
Semi-logarithmic plasma concentration–time curves of danofloxacin after oral administration of a 10 mg/kg dose in rainbow trout (*Oncorhynchus mykiss*) of different body sizes at 14 ± 0.5 °C (*n* = 6).

The plasma concentration–time curves and pharmacokinetic parameters of danofloxacin after oral administration of 10 mg/kg to rainbow trout of different body sizes are shown in Figure 1 and Table 1, respectively. Danofloxacin was detected in plasma up to 144 h in small- and medium-sized fish and up to 168 h in large-sized fish. In small-sized fish, the plasma t_1/2ʎz_, MRT_0–∞_, C_max_, AUC_0–last_, V_darea_/F, and CL/F were 27.42 h, 40.88 h, 2.53 µg/mL, 82.46 h·µg/mL, 4.65 L/kg, and 0.12 L/h/kg, respectively. Plasma t_1/2ʎz_, MRT_0–∞_, AUC_0–last_, and C_max_ increased with increasing trout size, whereas CL/F and V_darea_/F decreased. The T_max_ was similar in small, medium, and large sizes.

### 3.2. Tissue Pharmacokinetic Parameters

**Figure 2:**
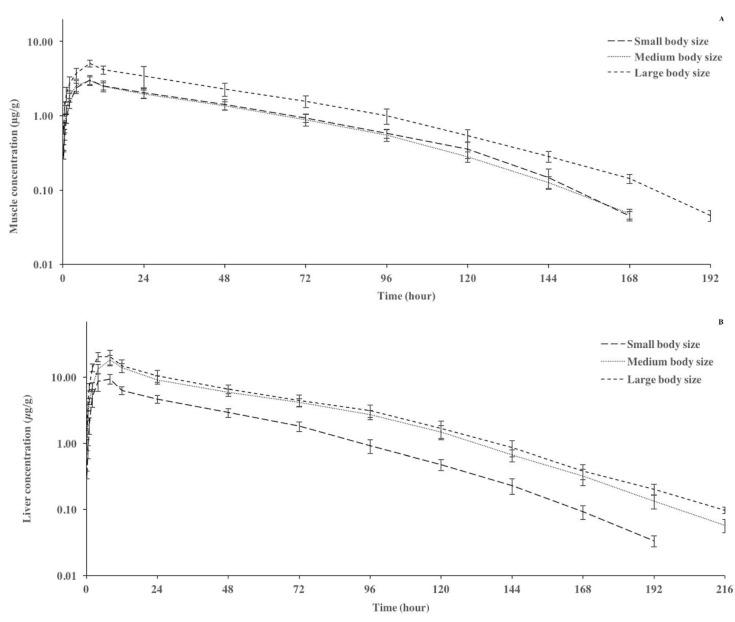

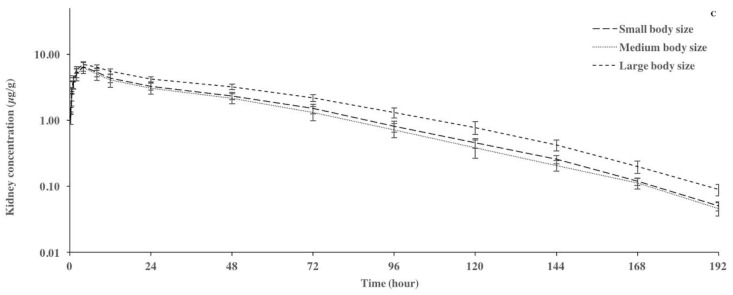
Semilogarithmic concentration–time curves of danofloxacin in muscle (**A**), liver (**B**), and kidney (**C**) after oral administration of a dose of 10 mg/kg in rainbow trout (*Oncorhynchus mykiss*) of different body sizes at 14 ± 0.5 °C (*n* = 6).

The tissue concentration–time curves and pharmacokinetic parameters of danofloxacin after oral administration of 10 mg/kg to rainbow trout of different body sizes are presented in Figure 2 and Table 2, respectively. The concentration of danofloxacin was observed in the following order: liver > kidney > muscle > plasma. The C_max_ and AUC_0–last_ values were significantly higher in large sizes than in small and medium sizes in all tissues. The AUC_0–last tissue_/AUC_0–last plasma_ ratios of danofloxacin in muscle, liver, and kidney tissues were 1.37–1.95, 4.33–6.76, and 2.22–3.34, respectively. The highest AUC_0–last tissue_/AUC_0–last plasma_ ratios for muscle, liver, and kidney were obtained in small-sized, medium-sized, and small-sized fish, respectively. The t_1/2ʎz_ and T_max_ values were quite close in all tissues of fish of different sizes.

## 4. Discussion

Age-related physiological changes can alter the pharmacokinetics of drugs, thereby affecting their therapeutic efficacy [30]. As environmental factors have a significant impact on fish growth, changes in fish size are more important than age. Although pharmacokinetic changes of danofloxacin have been reported in fish depending on species and route of administration [5,11,20,23,24,25,26], there is no information on size-related changes. In this study, pharmacokinetic changes of danofloxacin in rainbow trout of different body sizes were demonstrated for the first time. The plasma and tissue pharmacokinetics of danofloxacin were found to vary with body size.

The plasma t_1/2ʎz_, AUC_0-∞_, C_max_, and T_max_ values of danofloxacin in rainbow trout of different sizes (20–240 g) were 27.42–33.55 h, 85.07–174.43 h·µg/mL, 2.53–3.78 µg/mL, and 4 h, respectively, after oral administration of a dose of 10 mg/kg at 14 ± 0.5 °C. The values in this study were consistent with the plasma C_max_ (2.93 µg/mL), AUC_0-∞_ (144.46 h·µg/mL), and T_max_ (4 h) values reported after oral administration of 10 mg/kg to rainbow trout (105 ± 13 g) at 11.7 ± 0.8 °C, but different from the t_1/2ʎz_ value (41.04 h) [11]. The reported plasma t_1/2ʎz_, AUC_0–∞_, C_max_, and T_max_ values for the same dose and route of administration in goldfish (283 ± 53 g) at 20 °C were 47.79 h, 154.38 h·µg/mL, 3.23 µg/mL, and 2.73 h, respectively [24]. The oral t_1/2ʎz_ of danofloxacin in tilapia (71.5 ± 9.1 g), carp (380 g), and sea bass (180.73 ± 23.52 g) at 16–27 °C was between 16.87–47.7 h [5,20,25]. These differences in danofloxacin pharmacokinetics may be due to differences in fish species, body size, water temperature, drug formulation, and method of analysis.

The plasma V_darea_/F value of danofloxacin in trout of various sizes ranged from 2.77 to 4.65 L/kg. The V_d_ of danofloxacin was 2.28–2.55 L/kg after IV administration to trout [11,23] and 47 L/kg after oral administration to carp [20]. The lipophilic nature of danofloxacin and its low binding to plasma proteins contribute to its wide distribution volume, resulting in higher concentrations in tissue than in plasma [31,32]. V_darea_/F decreased from 4.65 to 2.77 L/kg as a result of the increase in fish body size. The V_d_ decreased from 84.20 to 49.69 L/kg for oxytetracycline [16] and increased from 2.10 to 3.24 L/kg for trifluralin [19] with increasing rainbow trout size. The peripheral distribution volume of di-2-ethylhexyl phthalate, a plasticizer, decreased with increasing rainbow trout size, while the central distribution volume varied [22]. These results show that the relationship between fish size and volume of distribution varies depending on the chemical used. Volume of distribution is influenced by changes in body composition and the extent of drug binding to plasma proteins [33]. It has been found that the binding ratio of chemicals to plasma proteins in fish can vary with body size [19,22]. However, the binding ratio of danofloxacin to plasma proteins did not change with age in calves [34]. Cardiac output, gill surface area, body composition (fat, protein, water), and organ weights vary with fish body size [18,19,22]. The ratio of muscle to fat increases as fish body size increases, while the ratio of organs such as liver, gut, and skin decreases [18,19]. The increase in adipose tissue with fish growth caused the increased V_d_ of trifluralin, which has a lipophilic structure [19]. Although danofloxacin has a lipophilic structure, its V_darea_/F decreased due to the increase in fish body size. In this study, danofloxacin was administered orally, but since bioavailability will not have an effect, the most appropriate way to determine V_d_ is by intravenous administration. The equation V_darea_/F = dose/concentration was used to calculate V_darea_/F. The change in V_darea_/F between groups may be due to the change in danofloxacin concentration and therefore bioavailability according to body size.

The CL/F of danofloxacin in small, medium, and large fish was 0.12, 0.09, and 0.06 L/h/kg, respectively. The CL/F decreased with increasing body size of the fish. Similarly, the CL/F of oxytetracycline and trifluralin decreased with increasing size of rainbow trout [16,19]. Danofloxacin is metabolised at different rates in different species and is then excreted in urine or bile [33]. Fluoroquinolones are also excreted through the gills [35]. As fish body size increases, metabolic activity and the relative weight of excretory organs such as the liver and gills decrease [16,18,19]. These changes may be the cause of the decrease in CL as a function of size. The t_1/2ʎz_ of danofloxacin was prolonged from 27.42 to 33.55 h depending on body size. In trout, similar results were reported for trifluralin [19], but the t_1/2ʎz_ of oxytetracycline was not affected by body size [16]. It has been suggested that the t_1/2ʎz_ of lipophilic and poorly metabolised chemicals may increase with increasing fish size [36].

After oral administration of 10 mg/kg danofloxacin, plasma C_max_ values were 2.53 ± 0.24, 3.05 ± 0.32, and 3.78 ± 0.38 µg/mL in small, medium, and large rainbow trout at 14 ± 0.5 °C, respectively. T_max_ was 4 h in all body sizes. After oral administration to various fish species at a dose of 10 mg/kg, plasma C_max_ and T_max_ values of 0.17–3.23 µg/mL and 2.73–10.6 h, respectively, have been reported [11,20,24,25]. The variability in plasma C_max_ and T_max_ of danofloxacin may be due to differences in fish species, body size, drug formulation, and water temperature. The plasma C_max_ and AUC values of the fish in this study increased as their body size increased. Similarly, the plasma C_max_ of oxytetracycline increased from 0.66 to 1.11 µg/mL and the plasma AUC_0–∞_ from 87.86 to 151.52 h·µg/mL with increasing body size in trout [16]. These results show that the absorption of the drug increases with increasing body size. The physiology (pH, etc.) of the digestive system depends on the life stage of the fish [37]. The extent of absorption, CL, and V_d_ of the drug are the factors that determine C_max_ and AUC [38]. The size-dependent change in plasma C_max_ and AUC may be due to differences in these parameters.

Following oral administration to rainbow trout at a dose of 10 mg/kg, danofloxacin was concentrated in the following order: liver > kidney > muscle > plasma. Tissue concentrations were found to be higher than plasma concentrations in studies in trout, koi, tilapia, and carp [23,25,26,31]. The highest concentrations of danofloxacin were found in the liver of trout and tilapia and in the kidney of carp and koi [25,26,31]. Danofloxacin is found in higher concentrations in the liver and kidney of fish, possibly due to the role of these organs in excretion. This ratio > 1 indicates that the drug has good tissue affinity in fish [39]. The AUC_tissue_/AUC_plasma_ ratios of danofloxacin in liver, kidney, and muscle of rainbow trout were greater than 1. Plasma and tissue C_max_ and AUC were higher in large fish compared to other body sizes. In large fish, the elevated concentrations of danofloxacin in tissues may be attributed to an increase in absorption extent and a decrease in Cl_T_. Furthermore, the concentration of danofloxacin in the liver and kidney of large fish increases because of its continuous elimination. The ratio AUC_tissue_/AUC_plasma_ showed that tissue penetration of danofloxacin was good, although it varied with fish body size, particularly in the kidney and liver. In addition, it was not linearly affected by changes in body size.

The minimum inhibitory concentration (MIC) of danofloxacin for susceptible bacteria was not determined in this study. However, the MIC values of danofloxacin for *Aeromonas hydrophila*, *Yersinia ruckeri*, and *Pseudomonas* spp. isolated from trout were 2–8 µg/mL, 0.02 µg/mL, and 1–3.2 µg/mL, respectively [11]. The effect of fluoroquinolones is concentration-dependent and pharmacokinetic/pharmacodynamic index parameters such as plasma AUC_0–24_/MIC and C_max_/MIC are used to assess their antibacterial activity. It is essential that these indices are maintained at appropriate levels to ensure therapeutic success and prevent the emergence of antibiotic resistance [4,40]. Danofloxacin was detectable for up to 144 h in small- and medium-sized fish and up to 168 h in large fish after a single dose. The plasma AUC_0–24_/MIC for long-acting antibiotics administered as a single dose cannot be directly compared to the values obtained in human medicine, which are usually for a duration exceeding 24 h [41]. In this case, a favoured method is to determine the scaling factor by dividing the plasma AUC_0–last_/MIC by the duration of the last detectable concentration [41]. In this study, the scaling factor following administration of danofloxacin at a dose of 10 mg/kg to small, medium, and large fish was 1.0 for bacteria with threshold MIC values of 0.57, 0.79 and 1.01 µg/mL, respectively. These results show that therapeutic efficacy increases with body size. However, the lack of a known optimal scaling factor for danofloxacin for bacteria isolated from fish precluded interpretation of the data from this study. A pharmacokinetic/pharmacodynamic study of danofloxacin is therefore required to determine the optimal scaling factor for the treatment of infections caused by susceptible bacteria in fish.

## 5. Conclusions

Fish body size caused significant changes in the plasma and tissue pharmacokinetics of danofloxacin. Plasma concentrations increased as the trout grew, but volume of distribution and elimination decreased. Danofloxacin concentrations in all tissues were higher than in plasma. Tissue penetration of danofloxacin is good, although the AUC_tissue_/AUC_plasma_ ratio varied with fish body size, but this difference was not linear. Danofloxacin administered at a dose of 10 mg/kg can maintain a mean plasma concentration equal to 1 × MIC for bacteria with threshold MIC values of 0.57, 0.79, and 1.01 µg/mL for 144 h in small and medium body sizes and 168 h in large body sizes. However, pharmacokinetic/pharmacodynamic studies of danofloxacin are required to determine the optimal therapeutic effect for the control of infections caused by susceptible bacteria in different body sizes of rainbow trout.

## Figures and Tables

**Table 1:** Plasma pharmacokinetic parameters of danofloxacin following oral administration of 10 mg/kg dose in rainbow trout (*Oncorhynchus mykiss*) of different body sizes at 14 ± 0.5 °C.

Parameters	Small Body Size	Medium Body Size	Large Body Size
t_1/2ʎz_ (h)	27.42	29.59	33.55
AUC_0–last_ (h·µg/mL)	82.46	113.26	169.43
AUC_0–∞_ (h·µg/mL)	85.07	117.39	174.43
AUC_extrap_ (%)	3.07	3.52	2.87
MRT_0–∞_ (h)	40.88	43.68	49.31
CL/F (L/h/kg)	0.12	0.09	0.06
V_darea_/F (L/kg)	4.65	3.64	2.77
C_max_ (µg/mL)	2.53 ± 0.24	3.05 ± 0.32	3.78 ± 0.38
T_max_ (h)	4	4	4

t_1/2ʎz_, elimination half-life; AUC, area under the concentration–time curve; AUC_extrap_%, area under the plasma concentration–time curve extrapolated from tlast to ∞ in % of the total AUC; MRT, mean residence time; CL/F, total body clearance; V_darea_/F, apparent volume of distribution; C_max_, peak plasma concentration; T_max_, time to reach the peak plasma concentration.

**Table 2:** Tissue pharmacokinetic parameters of danofloxacin following oral administration of a 10 mg/kg dose in rainbow trout (*Oncorhynchus mykiss*) of different body sizes at 14 ± 0.5 °C.

Parameters	Small Body Size	Medium Body Size	Large Body Size
**Muscle**			
t_1/2ʎz_ (h)	28.53	27.58	28.30
AUC_0–last_ (h·µg/g)	160.48	154.91	269.43
AUC_0–∞_ (h·µg/g)	162.13	156.90	271.47
AUC_extrap_ (%)	1.02	1.27	0.75
C_max_ (µg/g)	3.08 ± 0.36	3.08 ± 0.34	5.05 ± 0.54
T_max_ (h)	8	8	8
AUC_0–last muscle_/AUC_0–last plasma_	1.95	1.37	1.59
**Liver**			
t_1/2ʎz_ (h)	24.26	26.77	28.75
AUC_0–last_ (h·µg/g)	356.81	765.86	886.94
AUC_0–∞_ (h·µg/g)	357.86	768.18	891.08
AUC_extrap_ (%)	0.29	0.30	0.47
C_max_ (µg/g)	10.83 ± 0.77	18.81 ± 2.45	23.11 ± 3.39
T_max_ (h)	6	8	6
AUC_0–last liver_/AUC_0–last plasma_	4.33	6.76	5.23
**Kidney**			
t_1/2ʎz_ (h)	28.82	28.86	31.79
AUC_0–last_ (h·µg/g)	275.58	251.88	371.21
AUC_0–∞_ (h·µg/g)	277.66	253.96	375.34
AUC_extrap_ (%)	0.75	0.82	1.10
C_max_ (µg/g)	6.49 ± 0.98	6.52 ± 1.05	7.13 ± 0.50
T_max_ (h)	4	4	4
AUC_0–last kidney_/AUC_0–last plasma_	3.34	2.22	2.19

For abbreviations, see footnote of Table 1.

## Data Availability

The original contributions presented in this study are included in the article. Further inquiries can be directed to the corresponding authors.
